# Paracoccin Induces M1 Polarization of Macrophages via Interaction with TLR4

**DOI:** 10.3389/fmicb.2016.01003

**Published:** 2016-07-05

**Authors:** Mateus S. Freitas, Aline F. Oliveira, Thiago A. da Silva, Fabrício F. Fernandes, Relber A. Gonçales, Fausto Almeida, Maria C. Roque-Barreira

**Affiliations:** Departamento de Biologia Celular e Molecular e Bioagentes Patogênicos, Faculdade de Medicina de Ribeirão Preto, Universidade de São PauloRibeirão Preto, Brazil

**Keywords:** lectin, paracoccin, TLR, macrophage polarization, proinflammatory mediators

## Abstract

The fungal human pathogen *Paracoccidioides brasiliensis* contains paracoccin (PCN), a multi-domain protein that has lectin and *N*-acetyl-glucosaminidase activities, which account for its effects on the growth and morphogenesis of the fungus and on the activation of host macrophages through its interaction with TLR *N*-glycans. With the purpose of detailing the knowledge on the effects of PCN on macrophages, we used recombinant PCN expressed in *Pichia pastoris* (p-rPCN) to stimulate isolated murine peritoneal macrophages. The activation of these cells manifested through the release of high levels of inflammatory mediators, such as nitric oxide, TNF-α, IL-12p40, and IL-6. Furthermore, peritoneal macrophages stimulated with p-rPCN increased the relative expression of STAT1, SOCS3, and iNOS2 mRNA (M1 polarization markers). However, the expression of Arginase-1, Ym-1, and FIZZ1 (M2 polarization markers) remained at basal levels. Interestingly, the observed M1 macrophages’ polarization triggered by p-rPCN was abolished in cells obtained from knockout Toll-like receptor-4 mice. In this case, the p-rPCN-induced production of pro-inflammatory mediators was blocked too. These results demonstrate that the classical activation of macrophages induced by paracoccin depends on TLR4. Taken together, the results of our study indicate that paracoccin acts as a TLR agonist able to modulate immunity and exerts biological activities that favor its applicability as an immunotherapeutic agent to combat systemic fungal infections.

## Introduction

*Paracoccidioides brasiliensis* and *Paracoccidioides lutzii* are thermally dimorphic fungi and the causal agents of paracoccidioidomycosis (PCM), the most prevalent systemic mycosis in Latin America. The infection occurs through the inhalation of conidia, which convert into yeasts in the lungs, causing benign and transient lesions. It may progress into an acute form or, more frequently, reactivate later as a chronic and insidious disease (Restrepo, 2000; de Almeida, 2005; Laniado-Laborin, 2007), which disseminates to many different organs and tissues, particularly the skin, oral cavity, pharynx, larynx, upper gastrointestinal tract, lymph nodes, adrenal glands, and central nervous system (Tuder et al., 1985; Do Valle et al., 1993; Almeida et al., 2003; de Almeida, 2005; Restrepo et al., 2008).

The PCM course depends on factors inherent to the fungus, such as its virulence and antigenic composition, as well as on environmental conditions and the host’s immune state (Kurokawa et al., 2005). In this scenario, macrophages are essential in establishing the first barrier to the invading pathogens and in guiding the ensuing development of adaptive immunity (Hussell and Bell, 2014). Macrophages exhibit a high expression of pattern recognition receptors, especially Toll-like receptors (TLRs), whose interaction with agonists triggers cell activation. Macrophages can assume different types of activation depending on certain stimuli. “Classic” M1 macrophages are inflammatory cells that are involved in phagocytosis and killing of microbes, while “alternative” M2 cells favor angiogenesis, tissue remodeling, and repair (Murray and Wynn, 2011). The M1 and M2 subsets are discriminated by the production of nitric oxide (NO) and arginase activity, respectively, as well as by the expression of particular genes, such as iNOS2, STAT1, and SOCS3 for M1, and Arginase1, FIZZ1, YM1, STAT3, and SOCS1 for M2 (Lawrence and Natoli, 2011).

Our group has reported that *P. brasiliensis* yeast extracts contain an *N*-acetylglucosamine- and chitin-binding lectin called paracoccin (PCN) (Coltri et al., 2006). It exerts *N*-acetyl-β-D-glucosaminidase activity and is involved in fungal growth and morphogenesis (Ganiko et al., 2007; dos Reis Almeida et al., 2010; Dos Reis Almeida et al., 2011). More important for the biology of *P. brasiliensis*, PCN stimulates macrophages to produce high levels of NO and TNF-α (Coltri et al., 2006). Moreover, administration of a recombinant form of PCN expressed in *Escherichia coli* (herein named b-rPCN) confers protection against experimental PCM in a manner that depends on TLR2 and TLR4. This protection was primarily associated with the effects of b-rPCN on macrophages, triggered by its interaction with TLR *N*-glycans and culminating with the establishment of Th1 immunity (Alegre et al., 2014; Alegre-Maller et al., 2014).

*Pichia pastoris* (strain GS115) cells have been extensively used for the expression and large scale production of heterologous proteins (Mattanovich et al., 2012). In this study, we validated a recombinant form of PCN produced in *P. pastoris* (p-rPCN) to mimic the known features of the native protein and identified that the p-rPCN stimulus promotes M1 polarization of macrophages. We verified that this response depends heavily on the interaction between p-rPCN and TLR4.

## Materials and Methods

### Mice and Ethics Statement

Male C57BL/6 (wild-type, WT), TLR2 knockout (TLR2^-/-^), and TLR4 knockout (TLR4^-/-^) mice of 6–8 weeks of age were used. They were acquired from the vivarium on the campus of the University of São Paulo at Ribeirão Preto, São Paulo, Brazil, and housed in the animal facility of the Molecular and Cellular Biology Department, Faculty of Medicine of Ribeirão Preto, University of São Paulo, under optimized hygienic conditions. Animal procedures were approved by the Ethical Committee for Ethics in Animal Research (CETEA) of the School of Medicine at Ribeirão Preto, University of São Paulo, under protocol number 20/2013-1.

### Cloning, Expression in *Pichia pastoris*, and Purification of Recombinant Paracoccin

The full-length prediction encoding ORF for paracoccin, cloned in vector pUC57 by Alegre et al. (2014), was used as a template for PCR with forward primer 5′-CTCGAGATGGCGTTTGAAAACCAGATTG-3′ and reverse primer 5′-GCGGCCG CCCAGCTGCTGGTGCTAAAGC-3′, containing the restriction sites of the *XhoI* and *NotI* enzymes, respectively. The reaction was carried out in 30 cycles (30 s at 94°C, 30 s at 57°C, and 60 s at 72°C). The purified PCR product was cloned into the pGEM-T vector (Promega, Fitchburg, WI, USA), and the insert was removed from the vector with the aforementioned restriction enzymes and ligated into the pGAPzαA vector (Invitrogen, Carlsbad, CA, USA). The pGAPzαA-PCN vector was obtained and sequenced to determine the ligation success and the correct sequence of the insert. This vector was then linearized with the restriction enzyme *AvrII* so as to be used for the transformation of the *Pichia pastoris* GS115 strain, as described by Maleki et al. (2010). In short, 10 μg of the purified (using Illustra kit plasmidPrep Mini Spin – GE Healthcare, Little Chalfont, UK) and linearized plasmid were electroporated into the yeast in 0.2 cm cuvettes at 1.5 kV (25 μF and 200 Ω), using Gene Pulser (Bio-Rad, Hercules, CA, USA).

The transformants obtained on the selective YPD medium containing Zeocin (100 μg/mL) were confirmed by PCR. A clone was grown in 300 mL YPD liquid medium at 30°C, 220 rpm, for 72 h, for paracoccin recombinant expression and secretion. The culture supernatant was collected, dialyzed against phosphate buffered saline (PBS; 10 mM, pH 7.2), and concentrated 10 times using centrifugal filtration devices with a 10,000-molecular weight cut-off (Millipore, Darmstadt, Germany).

For paracoccin purification, the prepared culture supernatant was centrifuged at 10000 × *g*, at 5°C, and applied to a chitin column, manufactured as described by dos Reis Almeida et al. (2010), and equilibrated with PBS. After rinsing with 20 column volumes of PBS, the material adsorbed to chitin was eluted with 0.1% acetic acid. The eluate was concentrated and dialyzed against PBS. The product obtained from chromatography was analyzed by sodium dodecyl sulfate-polyacrylamide gel electrophoresis (SDS-PAGE) and submitted to quality control by assessing its lectin and enzymatic activities. The final product was named p-rPCN.

### Lectin Activity: Microplate Assay for p-rPCN Binding to Laminin

A high affinity polystyrene microplate (Corning Costar, Badhoevedorp, The Netherlands) was coated with laminin (250 ng/well) in carbonate-bicarbonate buffer (0.1 M; pH 9.6) overnight, at 4°C. After rinsing with PBS-Tween 20 (PBS-T) and blocking with PBS-T with 3% gelatin (blocking buffer) for 1 h at 37°C, rPCN (200 ng) or blocking buffer (negative control) was added to the wells in triplicates, and the microplates were incubated at room temperature for 2 h. After rinsing with PBS-T, IgY anti-rPCN antibodies (diluted 1:250 in PBS-T containing 1% gelatin) were added to the wells and incubated for 1 h at 37°C. After rinsing, murine biotinylated IgG anti-IgY (diluted to 1:1000 in PBS-T containing 1% gelatin) was added and incubated for 1 h at 37°C. As the last step, streptavidin-peroxidase conjugate (1:250) was added to each well for 1 h at room temperature. Following rinsing, the reaction was revealed by using the tetramethylbenzidine substrate (Pierce, Thermo Fisher Scientific Inc., Waltham, MA, USA), and color development was detected at a wavelength of 450 nm in a microplate reader (Power Wave X – BioTek Instruments, Inc., Winooski, VT, USA).

### Enzymatic Activity of p-rPCN

The *N*-acetyl-β-D-glucosaminidase activity of yeast recombinant paracoccin was assayed as previously described in detail (dos Reis Almeida et al., 2010; Almeida et al., 2015). The substrate ρ-nitrophenyl-*N*-acetyl-β-D-glucosaminide (100 μL, 5 mM; Sigma–Aldrich, St Louis, MO, USA) was mixed with 350 μL of 0.1 M sodium acetate (pH 5.5) and 5 μg of p-rPCN. As a negative control, we used the vehicle medium. The reaction was incubated for 16–18 h at 37°C, with 1 mL of 0.5 M sodium carbonate added. The enzyme activity values were determined by using a microplate reader at 405 nm (Power Wave X, BioTek Instruments, Inc.).

### Culture of Murine Peritoneal Macrophages: Stimulation with p-rPCN

Murine peritoneal macrophages were obtained from WT, TLR2^-/-^, and TLR4^-/-^ mice after an intraperitoneal injection with 1.0 mL of sterile 3% sodium thioglycollate (Sigma–Aldrich). After 3 days, the peritoneal cavity was washed with 5.0 mL of cold sterile PBS and the cells were harvested and centrifuged at 300 × *g* for 10 min. The cell pellet was resuspended in Dulbecco’s modified Eagle’s medium (Sigma–Aldrich) supplemented with 10% fetal bovine serum (HyClone, Logan, UT, USA) and 1% penicillin-streptomycin (Gibco, Thermo Fisher Scientific Inc.), and the cell counting was performed in a Neubauer chamber. The cells were plated in 48-well culture plates (1 × 10^6^ cells/mL) and incubated overnight at 37°C in a 5% CO_2_ atmosphere. The non-adherent cells were removed by gentle rinsing with PBS, while the adherent macrophages were incubated in Dulbecco’s modified Eagle’s medium supplemented with 10% fetal bovine serum. Elicited peritoneal macrophages were stimulated with p-rPCN (5.0 μg/mL). The positive controls were LPS (1 μg/mL) with IFN-γ (1 ng/mL) added for TLR2^-/-^ macrophages and Pam3CSK4 (100 ng/mL) for TLR4^-/-^ macrophages. The medium alone was used as a negative control. After variable incubation periods (specified in the legends), the culture supernatants were harvested and assessed for the concentration of mediators.

### Nitric Oxide Measurement

Nitric oxide concentration was inferred by measuring nitrite levels in the macrophage supernatants by using the Griess reagent system (Green et al., 1982). In short, 50 μL of the supernatants were distributed in 96-well microplates and incubated with 50 μL/well of Griess reagent (1.0% sulfanilamide, 0.1% naphthalenediamine dihydrochloride, and 2.5% H_3_PO_4_) at room temperature for 10 min. The absorbance at 550 nm was read using a Power Wave-X microplate reader (BioTek Instruments, Inc.). The absorbance was converted to micromolar (μM) NO on the basis of a standard curve, concomitantly generated by using known concentrations of NaNO_2_.

### Cytokine Measurement

Supernatants of stimulated macrophages were assessed for their levels of IL-12p40, IL-6, and TNF-α. The cytokines were detected by an enzyme-linked immunosorbent assay (ELISA) using an OptEIA kit (Pharmingen, San Diego, CA, USA), according to the manufacturer’s instructions. Standard curves allowed determining cytokine concentrations in pg/mL. The absorbance was read at 450 nm in the Power Wave-X microplate scanning spectrophotometer (BioTek Instruments, Inc.).

### Quantitative Reverse Transcription PCR

Macrophages (1 × 10^6^ cells/mL) from WT, TLR2^-/-^, and TLR4^-/-^ mice were incubated with rPCN (5 μg/mL), LPS (1 μg/mL), Pam3CSK4 (100 ng/mL), IFN-γ (1 ng/mL) plus IL-12p40 (50 ng/mL) (M1 macrophage inducers), IL-10 plus IL-4 (50 ng/mL both) (M2 macrophage inducers), or with medium alone, for 6 h. RNA from macrophages was extracted using Trizol Reagent (Invitrogen) according to the manufacturer’s instructions. The total RNA was reverse-transcribed into cDNA by the ImProm-II Reverse Transcription System (Promega) using oligo(dT). Quantitative real-time PCR was performed using SYBR Green (Applied Biosystems, Life Technologies, Carlsbad, CA, USA) on a 7500 Real-Time PCR System (Applied Biosystems). The relative expression of transcripts was quantified using the ΔΔCt method, and β-actin was used as endogenous control. The PCR primers used were: β-actin (S-CCTAAGGCCAACCGTGAAAA; AS-GAGGCATACAGGGACAGCACA), Ym1 (S-TCACAGGTCTGGCAATTCTTCTG; AS-ACTCCCTTCTATTGGCCTGTCC), Arginase-1 (S-GTTCCCAGATGTACCAGGATTC; AS-CGATGTCTTTGGCAGATATGC), FIZZ1 (S-CCTGAGATTCTGCCCCAGGAT; AS-TTCACTGGGACCATCAGCTGG), iNOS2 (S-CCGAAGCAAACATCACATTCA; AS-GGTCTAAAGGCTCCGGGCT), STAT1 (S-CACGCTGCCTATGATGTC; AS-CCTGGAGATTACGCTTGC), STAT3 (S-GGCACCTTGGATTGAGAG; AS-TGCTGATAGAGGACATTGG), SOCS1 (S-AGGATGGTAGCACGCAAC; AS-GAAGACGAGGACGAGGAG), and SOCS3 (S-AGGAGAGCGGATTCTACTG; AS-TCACACTGGATGCGTAGG).

### Statistical Analysis

The results are expressed as mean ± SEM. The statistical analysis was done using the *GraphPad Prism* software (GraphPad Software, San Diego, CA, USA). The homogeneous variance was analyzed, and the difference between means of groups was calculated by the analysis of variance (one-way) and Bonferroni’s test thereafter. Differences with *p* < 0.05 were considered statistically significant.

## Results

### Production of Recombinant Paracoccin in *Pichia pastoris*

**FIGURE 1:**
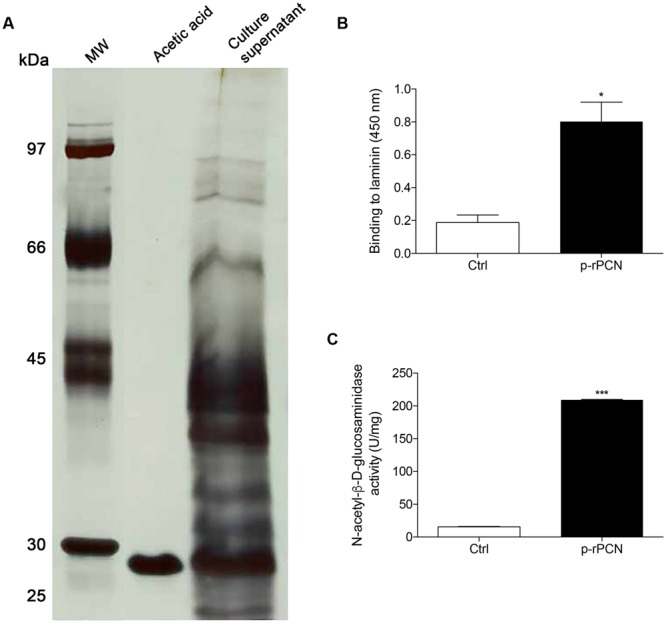
**Purification and biological activities of a recombinant form of paracoccin produced using the *Pichia pastoris* expression system. (A)** SDS-PAGE of the fraction of the culture supernatant that was adsorbed to chitin and eluted in acidic buffer; shows a 27 kDa silver stained band, as determined on the basis of the migration index of MM markers (lane MW). **(B)** Binding of the purified recombinant paracoccin (named p-rPCN, 200 ng) to the laminin coated wells (250 ng) of a microplate. The binding was revealed by reaction with anti-paracoccin IgY. **(C)** *N*-acetyl-glucosaminidase activity exerted by the p-rPCN as revealed using the substrate ρ-nitrophenyl-*N*-acetyl-β-D-glucosaminide (100 μL 5 mM ρ-NPGlcNAc). The results are expressed as mean ± SEM and were compared to the medium through one-way analysis of variance, followed by Bonferroni’s test. ^∗∗∗^*p* < 0.001 and ^∗^*p* < 0.05.

Paracoccin purified from *Paracoccidioides brasiliensis* has both lectin and enzymatic properties, which are mimicked by a recombinant form of the protein, expressed in *Escherichia coli* (b-rPCN) (Alegre et al., 2014). To be stricter concerning the required absence of LPS in our preparations, we expressed paracoccin in the *P. pastoris* strain GS115, transformed with the pGAPzαA vector and containing the synthetic transcript of this protein. The secreted protein was purified in a chitin column and called p-rPCN. The electrophoretic profile of the bound fraction showed that it consists of a single 27 kDa band (**Figure 1A**), which is the expected molecular mass for paracoccin. Our recombinant PCN exhibited the properties of the native protein, which was evident from several facts. First, the fact that its purification was founded on binding to chitin showed that p-rPCN retains the carbohydrate binding capacity of the native protein. Second, it could be detected by binding to laminin (**Figure 1B**), a property that depends on the lectin domain of paracoccin, as previously reported for PCN and b-rPCN. Finally, using the substrate ρ-nitrophenyl-*N*-acetyl-β-D-glucosaminide, we demonstrated that p-rPCN exhibits *N*-acetyl-β-D-glucosaminidase activity (**Figure 1C**). These results validated the use of p-rPCN in the present study aiming to detail the paracoccin effects exerted on macrophages.

### Recombinant PCN Induces Macrophages to Produce Proinflammatory Mediators

**FIGURE 2:**
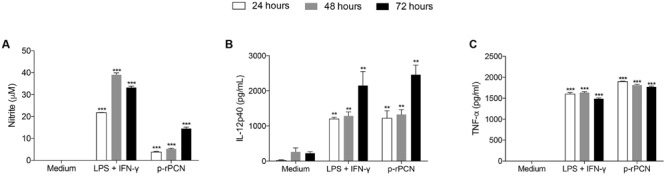
**p-rPCN induces macrophages to release proinflammatory mediators.** Peritoneal macrophages (1 × 10^6^ cells/mL) from WT mice were stimulated with p-rPCN (5 μg/mL) for 24, 48, and 72 h. The culture supernatants were assessed for nitrite concentration (μM), measured by the standard Griess reaction **(A)**, IL-12p40 **(B)** and TNF-α **(C)** levels (pg/mL) using ELISA. The medium and LPS (1 μg/mL) plus IFN-γ (1 ng/mL) were used as negative and positive controls, respectively. The results are expressed as mean ± SEM and were compared to the medium through one-way analysis of variance, followed by Bonferroni’s test. ^∗∗∗^*p* < 0.001 and ^∗∗^*p* < 0.01.

We have previously demonstrated that native PCN and b-rPCN induce mouse peritoneal macrophages to release high levels of TNF-α and NO (Coltri et al., 2006; Alegre et al., 2014). Then, we examined whether p-rPCN could also stimulate mouse peritoneal macrophages to release inflammatory mediators. Indeed, p-rPCN induced the release of significant levels of NO, IL-12p40, and TNF-α in macrophages (**Figures 2A–C**, respectively) at 24, 48, and 72 h after the stimulus. The time-course production of IL-12 (**Figure 2B**) and TNF-α (**Figure 2C**) was close to that determined by the positive control and consisted of a mixture of LPS and IFN-γ. These findings demonstrate that p-rPCN induces macrophages to release proinflammatory cytokines.

### Paracoccin Induces M1 Macrophage Polarization

**FIGURE 3:**
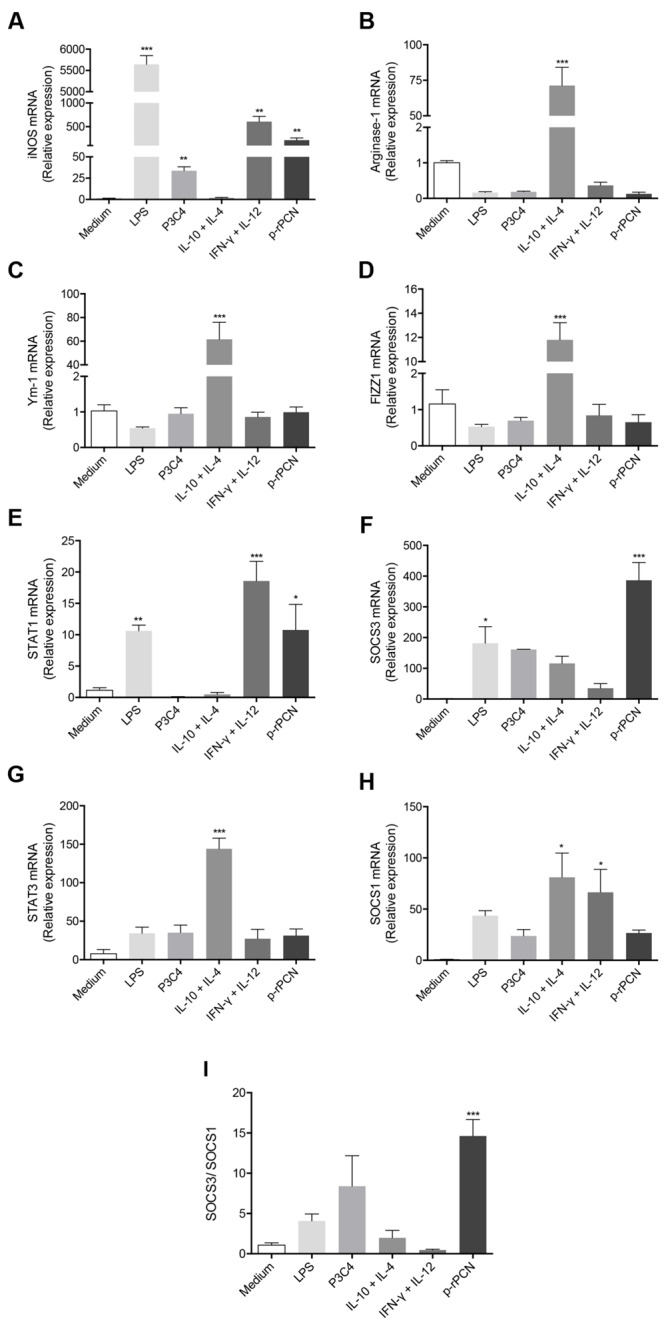
**Paracoccin induces classical activation of macrophages.** Murine peritoneal macrophages from WT (1 × 10^6^ cells/mL) mice were stimulated with p-rPCN (5 μg/mL) for 6 h. As positive controls for the classical activation, the following inducers were used: LPS (1 μg/mL), Pam3CSK4 (100 ng/mL), and IFN-γ (1 ng/mL) plus IL-12p40 (50 ng/mL). For alternative activation, IL-10 plus IL-4 (50 ng/mL both) was used. The medium alone was used as a negative control. After extraction using TRizol Reagent^®^, the RNA was converted into cDNA and the expression of iNOS **(A)**, Arginase-1 **(B)**, Ym-1 **(C)**, FIZZ1 **(D)**, STAT1 **(E)**, SOCS3 **(F)**, STAT3 **(G)**, and SOCS1 **(H)** were analyzed by real-time PCR. The ratio SOCS3/SOCS1 **(I)** was calculated. The relative expression was determined as described in Section “Materials and Methods,” and the results obtained under stimulus were compared to those obtained using the medium. Data are shown in mean ± SEM and were compared through one-way analysis of variance, followed by Bonferroni’s test. ^∗∗∗^*p* < 0.001, ^∗∗^*p* < 0.01, and ^∗^*p* < 0.05.

To advance in the study of the effects of paracoccin on macrophage activation, using real-time PCR, we quantified the expression of M1 (iNOS2) and M2 (Arginase-1, Ym-1, and FIZZ1) polarization markers in response to p-rPCN. Peritoneal macrophages stimulated with p-rPCN showed a more than 200-fold increase in iNOS mRNA, attaining levels that were similar to those in macrophages stimulated by IFN-γ plus IL-12 (**Figure 3A**). The analysis of M2 markers revealed that the p-rPCN stimulus did not change Arginase-1, Ym-1, and FIZZ1 expression, whose levels remained close to those found in unstimulated cells and were significantly lower than those measured by the IL-10 plus IL-4 control stimulus (**Figures 3B–D**). In addition, the expression measurement of key signaling mediators associated with M1/M2 dichotomy showed that the levels of STAT1 and SOCS3 mRNA increased by 10- and 380-fold, respectively, in response to the p-rPCN stimulus (**Figures 3E,F**). On the other hand, this stimulus did not affect the expression of STAT3 and SOCS1 (**Figures 3G,H**). Furthermore, the ratio of the expression of SOCS3 and SOCS1 increased 15-fold in response to p-rPCN (**Figure 3I**). These results indicate that p-rPCN induces the classical activation of macrophages, with the involvement of STAT1 and SOCS3.

### TLR4 Is Crucial for the Macrophage Release of Proinflammatory Cytokines Induced by Paracoccin

**FIGURE 4:**
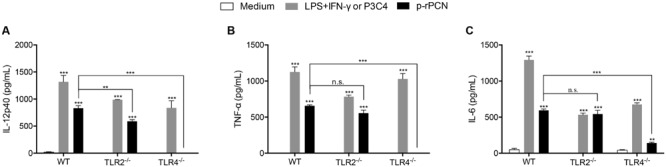
**Involvement of TLR2 and TLR4 in the proinflammatory cytokine production induced by paracoccin.** Macrophages (1 × 10^6^ cells/mL) obtained from WT, TLR2^-/-^, and TLR4^-/-^ mice were stimulated for 24 h by p-rPCN (5 μg/mL). A mixture of LPS (1 μg/mL) and IFN-γ (1 ng/mL) served as the positive control for WT and TLR2^-/-^ macrophages; Pam3CSK4 (100 ng/mL) was used as the positive control for TLR4^-/-^ macrophages. The culture supernatants were assessed for the levels of IL-12p40 **(A)**, TNF-α **(B)**, and IL-6 **(C)** using ELISA. The values (pg/mL) were expressed in mean ± SEM and compared to the negative control (the medium) through one-way analysis of variance, followed by Bonferroni’s test. ^∗∗∗^*p* < 0.001, ^∗∗^*p* < 0.01, and non-significant differences (n.s.).

Given that recombinant paracoccin expressed in *E. coli* interacts with the *N*-glycans of TLR2 and TLR4 on antigen-presenting cells and promotes IL-12 production (Alegre-Maller et al., 2014), we examined the relevance of TLR2 and TLR4 in the production of inflammatory mediators induced by p-rPCN. We stimulated peritoneal macrophages obtained from WT, TLR2^-/-^, or TLR4^-/-^ mice with p-rPCN for 48 h and quantified the levels of IL-12p40, TNF-α, and IL-6 in the cell supernatants. We found that the lack of TLR2 diminished the IL-12p40 production induced by p-rPCN compared to that of WT macrophages, while the TNF-α and IL-6 levels were unaffected. The absence of TLR4, in turn, abolished the p-rPCN-induced production of IL-12p40 and TNF-α (**Figures 4A,B**) and severely impaired the IL-6 release (**Figure 4C**). Interestingly, the cytokine production induced by the control agonists of either receptor (Pam3CSK4 for TLR2 and LPS plus IFN-γ for TLR4) was inhibited by the absence of the respective TLR, but less affected than the production promoted by p-rPCN. We conclude that TLR2 and TLR4 participate in the induction of proinflammatory cytokine production in macrophages stimulated by paracoccin; a more notable role is attributed to TLR4.

### TLR4 Is Crucial for the Classical Polarization of Macrophages Induced by Paracoccin

**FIGURE 5:**
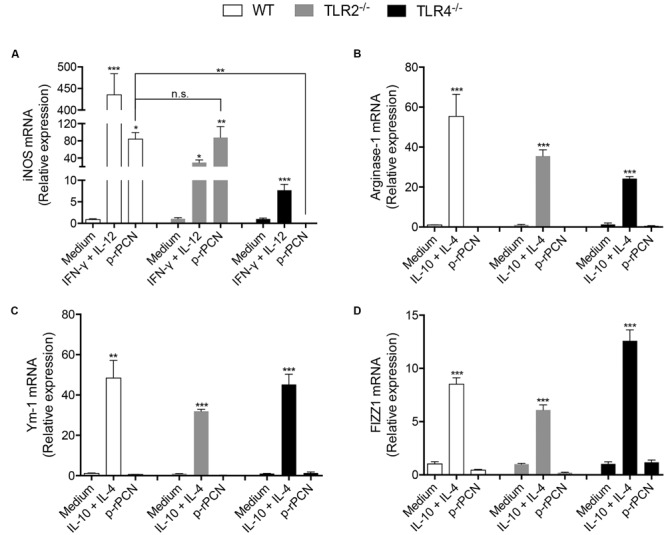
**Involvement of TLR4 in the classical activation of macrophages by paracoccin.** Macrophages (1 × 10^6^ cells/mL) from WT, TLR2^-/-^, and TLR4^-/-^ were stimulated for 6 h with p-rPCN (5 μg/mL). As a positive control for the classical activation, the mixture of IFN-γ (1 ng/mL) and IL-12p40 (50 ng/mL) was used. For alternative activation, a mixture of IL-10 plus IL-4 (50 ng/mL both) was used. The medium alone was used as the negative control. After extraction using TRizol Reagent^®^, the RNA was converted into cDNA and the expression of iNOS **(A)**, Arginase-1 **(B)**, Ym-1 **(C)**, and FIZZ1 **(D)** were analyzed by real-time PCR. The relative expressions were determined as described in Section “Materials and Methods” and compared between WT, TLR2^-/-^ and TLR4^-/-^ macrophages. The results are expressed in mean ± SEM and were compared through one-way analysis of variance, followed by Bonferroni’s test. ^∗∗∗^*p* < 0.001, ^∗∗^*p* < 0.01, ^∗^*p* < 0.05, and non-significant differences (n.s).

To expand the investigation of the roles of TLR2 and TLR4 on the effects of paracoccin on macrophages, we analyzed whether the M1 polarization of these cells promoted by p-rPCN could be affected by the absence of TLR2 or TLR4. Following the stimulation of peritoneal macrophages harvested from WT, TLR2^-/-^, or TLR4^-/-^ mice, the cells were assessed for the expression of iNOS, Arginase-1, Ym-1, and FIZZ1. The iNOS expression stimulated by p-rPCN was not affected by the absence of TLR2, but was blocked in TLR4^-/-^ macrophages (**Figure 5A**). The expression of M2 polarization markers, which did not respond to the p-rPCN stimulus, remained in cells from TLR2^-/-^ or TLR4^-/-^ mice at levels as low as those verified in non-stimulated macrophages (medium) (**Figures 5B–D**). Therefore, we conclude that TLR4 is critical for the paracoccin-induced M1 polarization of macrophages.

## Discussion

Considering the previous demonstration that the interaction of paracoccin with TLR2 and TLR4 on macrophages modulates immunity and positively interferes in the PCM course (Alegre et al., 2014; Alegre-Maller et al., 2014), herein we detailed additional events triggered by paracoccin in those cells. By using a recombinant form expressed in *P. pastoris* (p-rPCN), we showed that paracoccin induces M1 polarization of macrophages, as well as the production of inflammatory mediators. Notably, TLR4 was critical in the establishment of these responses, suggesting that their triggering was due to the interaction of paracoccin with TLR4 *N*-glycans, as was previously reported to happen with TLR2 (Alegre-Maller et al., 2014). We postulate that the events reported herein account for the protective effect of paracoccin administration against *P. brasiliensis* infection.

The expression of paracoccin in *Pichia pastoris* was advantageous compared to its expression in *E. coli*, which was utilized in our previous studies. The expression in *P. pastoris* allowed us to avoid several exhaustive chromatographic procedures used to remove bacterial endotoxins during the recombinant protein purification. In addition, it provided substantially higher final yields of homogeneous recombinant paracoccin. Besides, the potential prejudice in protein activity, usually resulting from the hyperglycosylation of proteins expressed in *Pichia pastoris*, did not occur, as demonstrated by the detection of preserved lectin and enzymatic activities and by the fact that there was no shift in the MM of the protein in the SDS-PAGE analysis (Macauley-Patrick et al., 2005; Mattanovich et al., 2012).

Once validated as able to mimic the effects of previously used preparations, p-rPCN was used to investigate additional events implicated in the effects of paracoccin on macrophage activation. As a manifestation of this phenomenon, we found that macrophages produced high levels of inflammatory mediators in response to the p-rPCN stimulus, just as they did in response to the native protein (Coltri et al., 2006). This is an essential activity, considering that we envisage paracoccin to become available for pharmaceutical application as an immunomodulatory agent capable of conferring protection against deep fungal diseases by favoring the development of a balanced Th1 immunity (Alegre et al., 2014; Alegre-Maller et al., 2014). Indeed, the immunomodulatory activity triggered by pathogen lectins is widely studied, and we found strong evidence that *Toxoplasma gondii* microneme proteins, which exhibit lectin properties, confer protection against experimental toxoplasmosis (Pinzan et al., 2015).

We previously verified that the mechanisms of protection conferred by paracoccin against murine PCM require the participation of innate immunity cells (Alegre-Maller et al., 2014). Macrophages and dendritic cells are responsible for the initial response in *P. brasiliensis* infection, and macrophage polarization is known to differ between resistant and susceptible mice infected with the fungus (Calich et al., 2008; Pina et al., 2008; Feriotti et al., 2013). We then investigated the ability of p-rPCN to induce macrophage polarization. The relative expression of M1 and M2 markers showed that p-rPCN stimulated an increase in the expression of iNOS, STAT1, and SOCS3 mRNA, indicating the occurrence of M1 polarization. The activation of STAT1 and STAT3 finely regulates macrophage polarization and activities, and the NF-κB and STAT1 activation induces M1 macrophage polarization (Sica and Mantovani, 2012). The occurrence of M1 polarization is consistent with the augmented production of proinflammatory cytokines by the p-rPCN-stimulated macrophages. It was already demonstrated that M1 polarization favors the innate mechanism of *P. brasiliensis* elimination (Pina et al., 2008; Feriotti et al., 2013), as well as contributing to fungal clearance during experimental infection with *Cryptococcus neoformans* (Hardison et al., 2010; Davis et al., 2013). On the other hand, M2 polarization is related to fungal dissemination. Therefore, we suggest that, in addition to the previously reported effects of paracoccin administration to infected hosts, these include M1 polarization, which contributes to the verified PCM control. This scenario goes in favor of the idea that paracoccin can be applied as an immunotherapeutic agent against PCM. The mechanism behind the mentioned events typically involves TLRs and their ligands. We found that TLR2 participates in cytokine production induced by p-rPCN on macrophages and that TLR4 is required not only to induce the release of proinflammatory cytokines, but also to promote the M1 polarization of macrophages in response to p-rPCN. The interaction of paracoccin with TLR2 and TLR4 *N*-glycans has already been shown to account for the activation of macrophages and for *P. brasiliensis* elimination. However, there are few studies on the effects of TLR2 and TLR4 activation during the course of this fungal infection. TLR2-deficient mice infected with *P. brasiliensis* showed increased Th17 immunity, reduced regulatory T cells, and unrestrained inflammatory pulmonary reactions (Loures et al., 2009). On the other hand, TLR4-defective mice infected with *P. brasiliensis* exhibited reduced fungal burdens and lower levels of inflammatory mediators compared to TLR4-competent mice (Loures et al., 2010). This observation partially supports our data regarding the critical role of TLR4 in the response to the p-rPCN stimulus.

The ability of b-rPCN to interact with TLR2 and TLR4 on APCs and the therapeutic effects triggered by such interactions on the course of experimental PCM were already established (Alegre et al., 2014; Alegre-Maller et al., 2014). Our current results reinforce the idea that paracoccin administration can be useful in the therapy of PCM and demonstrate that TLR4 is the main receptor responsible for the biological activities of macrophages triggered by p-rPCN. Therefore, paracoccin can be considered a novel TLR agonist that modulates the host immune response through carbohydrate recognition.

## Author Contributions

Conceived and designed the experiments: MF, AO, TS, FF, RG, FA, MR-B. Performed the experiments: MF, AO, TS, FF. Analyzed the data: MF, AO, TS, FF. Wrote the paper: MF, AO, TS, FF, FA, MR-B. All authors have read and approved the final manuscript.

## Conflict of Interest Statement

The authors declare that the research was conducted in the absence of any commercial or financial relationships that could be construed as a potential conflict of interest.
